# Cone-Beam Computed Tomography Comparison of Canal Transportation after Preparation with BioRaCe and Mtwo Rotary Instruments and Hand K-Flexofiles

**Published:** 2014-07-05

**Authors:** Hadi Mokhtari, Mahdi Niknami, Aydin Sohrabi, Ehsan Habibivand, Hamid Reza Mokhtari Zonouzi, Saeed Rahimi, Vahid Zand

**Affiliations:** a Dental and Periodontal Research Center, Department of Endodontics, Faculty of Dentistry, Tabriz University of Medical Sciences, Tabriz, Iran; b Department of Oral and Maxillofacial Radiology, Faculty of Dentistry, Tehran University of Medical Sciences, Tehran, Iran; c Dental and Periodontal Research Center, Department of Orthodontics, Faculty of Dentistry, Tabriz University of Medical Sciences, Tabriz, Iran; d Department of Endodontics, Faculty of Dentistry, Tabriz University of Medical Sciences, Tabriz, Iran

**Keywords:** BioRaCe, Canal Transportation, CBCT, Cone-Beam Computed Tomography, Mtwo; Root Canal Preparation

## Abstract

**Introduction:** The aim of this *in vitro* study was to evaluate the transportation of mesiobuccal canals of mandibular first molars prepared with either BioRaCe or Mtwo rotary instruments or hand K-Flexofile, by means of cone-beam computed tomography (CBCT). **Methods and Materials:** Forty-five mandibular molars were selected and randomly divided into three groups (*n*=15). Mesiobuccal roots of these teeth were prepared by BioRaCe, Mtwo, or hand K-Flexofile. Transportation was measured by pre- and post-operative CBCT images. Two-way ANOVA analysis was applied to detect any differences between the groups followed by the post hoc Tukey’s tests. The level of significance was set at 0.05.** Results: **The non-parametric Friedman test was used to compare the behavior of each file at 3-, 6- and 9-mm levels. There were no significant differences between different levels in Mtwo group (*P*=0.15); however, the differences in K-Flexofile and BioRaCe groups were significant (*P*>0.05). The post hoc Tukey’s test revealed significant differences between BioRaCe and K-Flexofile and also between Mtwo and K-Flexofile, both in the 3-mm depths (*P*<0.05).** Conclusion:** Under the limitations of the present study, BioRaCe and Mtwo rotary instruments are considered suitable for canal preparation to greater apical sizes provided that the recommended sequences are observed.

## Introduction

Preparation and debridement of the root canal system is the most important step of root canal treatment (RCT). The procedure involves enlarging and shaping of the root canal(s) along with maintaining the anatomy of the apical foramen [1]. These procedures are potentially associated with some difficulties and mishaps in curved canals, with canal transportation being one of the most common ones [2]. Despite introduction of various techniques to prevent such problems, there are still obstacles to proper preparation of curved canals [3, 4]. Introduction of nickel-titanium (NiTi) files by Walia *et al.* [5] and the subsequent development of rotary systems made it possible to prepare curved canals more easily and properly.

Mtwo rotary files (VDW, Munich, Germany) have an S-shaped cross-section with deep cutting edges and low radial contact to increase their flexibility and improve the file performance inside the root canal. Their superiority to other files has been evaluated from various aspects and is confirmed by several studies [6-8].

**Figure 1:**
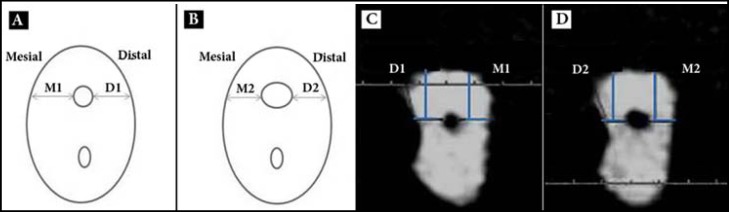
*A, B*) Schematic; and *C, D)* CBCT view of pre- and post-operative cross-sections of mesiobuccal canal; evaluation of canal transportation was done according to the following formula: (M1-M2)-(D1-D2)

On the other hand, some studies have shown that larger apical preparation is an important factor in proper removal of the bacteria from the canal walls. These studies showed that bacterial count in the root canals significantly decreased with an increase in the size of apical preparation [9, 10]. Therefore, aiming at increasing the apical size, BioRaCe rotary files (FKG Dentaire, La-Chaux-de Fonds, Switzerland) were introduced. Despite structural similarities with RaCe files (non-cutting safety tip and a triangular cross-section with alternating cutting edges), they are different in size, taper and the sequence of use. According to the manufacturer, the chief aim of designing these files was to achieve a greater apical size in order to attain better debridement with fewer files and errors [6, 11, 12]. However, a study by Buchanan [13] showed that the amount of canal transportation increases with apical preparation greater than size 25.

Various techniques have been used to evaluate the performance of files inside the root canals, including longitudinal and transverse sectioning and use of radiographs [14, 15]. Computed tomography (CT) techniques have been proposed due to their non-destructive nature and the possibility of measuring the amount of dentin removed from the root canal walls [16]. Cone-beam computed tomography (CBCT), provides a three-dimensional image with high accuracy and quality and thus facilitates a proper evaluation of canal preparation [17-19].

To date no studies have used CBCT scanning for evaluation of transportation in the mesiobuccal (MB) canal of mandibular first permanent molars prepared with either BioRaCe or Mtwo nickel-titanium (Ni-Ti) rotary instruments, or hand K-Flexofile. Therefore, the aim of the present study was to evaluate the transportation of MB canal of mandibular first permanent molars prepared with either of the aforementioned files by means of CBCT.

## Methods and Materials

***Sample selection***

Forty-five mandibular first molars extracted for periodontal reasons were selected for this study. All the samples had mature apical foramina and their MB root canals were uncalcified, confirmed by an initial radiograph. The curvature of the canals was determined using a periapical radiograph according to the method introduced by Schneider [20]. The AutoCAD 2007 software program (Autodesk, Inc., Mill Valley, California, USA) was used to determine the radius of curvature, as previously explained by Pruett *et al.* [21]. Teeth with root curvatures of 22 to 40 degrees and curvature radii of 5.5 to 9.9 mm were selected for canal preparation.

***Sample preparation***

All the teeth were stored in normal saline solution at 4^°^ C until the start of the procedure. The lengths of the specimens were reduced to 18 mm from the anatomic apex to coronal area by cutting the crowns away. The samples were randomly divided into 3 groups (*n*=15). All samples were numbered and mounted in laboratory putty.

Initially, a #10 or 15 K-file (Dentsply, Maillefer, Switzerland) was placed in each root canal with minimal pressure to make sure of an open path in the canal. Working length (WL) was established at 1 mm short of the apical foramen with a #10 K-file. Then the following protocols were used in the three groups:

***Group 1: ***BioRaCe files (FKG Dentaire, La-Chaux-de Fonds, Switzerland) were used in the following sequence to prepare the samples; BR0; 25/0.08, BR1; 15/0.05, BR2; 25/0.04, BR3; 25/0.06, BR4; 35/0.04, and BR5; 40/0.04. Files were installed on an electric handpiece (TCM Endo; Nouvag, Goldach, Switzerand) set at speed of 500 rpm and 1 Nm torque, according to the manufacturer’s instructions. Preparation was done with crown-down technique.

***Group 2:*** Mtwo files (VDW, Munich, Germany) were used in the following sequence: 10/0.04; 15/0.05; 20/0.06; 25/0.06; 30/0.05; 35/0.04; and 40/0.04, at a speed of 280 rpm and 3 Nm torque. Preparation was made using single-length technique.

***Group 3:*** Hand K-Flexofiles (Dentsply, Maillefer, Ballaigues, Switzerland) were used in the following sequence to prepare the canals: 15, 20, 25, 30, 35, and 40. Preparation was made with single-length technique. In all of the groups, after each file, a K-file was used to the WL for recapitulation. During preparation with each file, 2 mL of 2.5% sodium hypochlorite (NaOCl) and 2 mL of 17% ethylenediaminetetraacetic acid (EDTA) were used for irrigation.

**Table 1:** Descriptive statistics of canal transportation after instrumentation

	**3 mm**		**6 mm**		**9 mm**	
	Mean (SD)	Median	Mean (SD)	Median	Mean (SD)	Median
**BioRaCe**		0.06 (0.10)^ a1^	0.00	0.01 (0.04)^b1^	0.00	0.12 (0.19)^ a1^	0.00
**Mtwo**		0.08 (0.08)^ a1^	0.16	0.14 (0.12)^ a2^	0.16	0.08 (0.13^ )a1^	0.00
**K-Flexofile**		0.17 (0.07)^a2^	0.16	0.10 (0.09)^b2^	0.16	0.21 (0.13)^a2^	0.16

*Similar letters in each row indicate statistically non-significant differences; similar digits in each column indicate statistically non-significant differences*

***Imaging and analysis of images***

The CBCT machine, NewTom VGi (QR SRL Co., Verona, Italy) was used in two stages (preparation up to #25 and preparation up to #40) to prepare each series of radiographs with a background of 8×8 cm, 110 kVp, 19 mA and 5.4 sec of exposure time. The NTT Viewer software (NTT Software Corporation, Yokohama, Japan) was used to evaluate the axial radiographs of the apical area at 3-, 6- and 9-mm intervals.

***Evaluation of canal transportation***

Evaluation of canal transportation by different files, was carried out according to the technique described by Gambill *et al.* [22], using the following formula:[(M1-M2)-(D1-D2)].

According to Figure 1, M1 is the distance between the external surface of the mesial aspect of the root and the mesial wall of the prepared portion of the canal up to file #25. M2 is the distance between the external surface of the mesial aspect of the root and the mesial wall of the prepared portion of the canal up to file #40. D1 is the distance between the external surface of the distal aspect of the root and the distal wall of the prepared portion of the canal up to file #25. D2 is the distance between the external surface of the distal aspect of the root and the distal wall of the prepared portion of the canal up to file #40. The amount of transportation in each canal was assessed at three different sections of the canal (3-, 6- and 9-mm from the apex).

In the formulas above a value close to “1” indicates the greatest amount of canal transportation and a value close to “zero” indicates the least amount of canal transportation.

## Results

Canal transportation in the three groups (BioRaCe, Mtwo, and K-Flexofile) and at three different sections of the canals (3, 6 and 9 mm from apex) are presented in Table 1. Data distribution in groups was not normal. Non-parametric Friedman test was used to compare the behavior of each file between the 3-, 6- and 9-mm levels. There were nonsignificant differences between different levels in Mtwo files; however, such a difference existed in BioRaCe and K-Flexofiles. The Wilcoxon’s signed rank test was used to compare different levels in BioRaCe files, demonstrating differences between 6-mm and 9-mm levels (*P*=0.026); however, there were no significant differences between 3- and 6-mm levels and between 3- and 9-mm levels (*P*=0.06, *P*=0.23, respectively). The Wilcoxon’s signed rank test was used to compare different levels in K-Flexofile group, demonstrating differences between 3- and 6-mm level and between 6- and 9-mm levels (*P*=0.014, *P*=0.035, respectively); however, there were no significant differences between 3-mm and 9-mm levels (*P*=0.15). The non-parametric Kruskal‒Wallis test was used to compare the performance of the three different files at the same level. The results showed differences between the files at all the levels. The Mann‒Whitney U-test was used for two-by-two comparison of canals at 3-mm level. The results showed that the amount of transportation in BioRaCe and Mtwo files were different from the K-Flexofiles (*P*=0.004 and *P*=0.008), with no significant differences between BioRaCe and Mtwo files (*P*=0.661).

The results at 6-mm level showed that BioRaCe-induced canal transportation was different from the other two groups, *i.e*. K-Flexofile and Mtwo files (*P*=0.002 and *P*=0.001), with no significant differences between Mtwo and K-Flexofiles (*P*=0.355).

Mann‒Whitney U-test showed that at 9-mm level, K-Flexofile was different from Mtwo and BioRaCe (*P*=0.012 and *P*=0.03, respectively), with no differences between Mtwo and BioRaCe (*P*=0.629) (Table 1).

## Discussion

One of the most important aims of root canal treatment is to produce a conical canal shape from the apical to the coronal area, thus preserving the original general shape of the canal [23]. This is difficult to achieve in curved root canals and some errors might occur during the process, including canal transportation, ledge formation and/or canal perforation [2, 19]. Various techniques have been proposed to evaluate the files used during RCT, preparation techniques and finally the quality of root canal preparation so that the canal shape before and after mechanical preparation can be compared. These techniques include conventional radiography, root sectioning, *etc.* Conventional radiographic techniques provide a two-dimensional image of an object and such techniques cannot provide transverse sections [24]. The most commonly used technique is the cross-sectioning technique which is thoroughly destructive and usually results in the loss of specimens [19, 25]. CBCT imaging technique is known as a non-invasive technique for the evaluation of root canal anatomy before and after root canal preparation. It has been shown that use of this technique for evaluating the quality of root canal preparation yields better results compared to other techniques [26, 27].

Bacteria and their byproducts are major etiologic factors in endodontic diseases. Prevention or reduction of root canal bacterial contamination is the main aim of endodontic treatment [28-30]. In a study by Khademi *et al.* [31] the amount of apical preparation for the penetration of canal irrigants to the apical third of the canals was reported to be at least #30. Another study showed a significant decrease in bacterial counts of the infected canal dentin with an increase in apical preparation size from #30 to #40 [28]. Rollison *et al.* [29] reported that debridement of infected canals with a #50 file is better achieved compared to #30 file.

According to the statement of manufacturer, the major goal of BioRaCe sequence is to achieve apical preparation sizes that are scientifically proven to effectively disinfect the canal [6, 11, 12]. Bonaccorso *et al.* [6] evaluated canal transportation with BioRaCe files in prefabricated S-shaped resin canals under a stereomicroscope. Their reported results were similar to those of the present study. Pasternak-Junior *et al.* [30] evaluated canal transportation during preparation with RaCe files with the use of CBCT. They reported no significant differences in canal preparation from file #35 to #50. Canal displacement in the present study with BioRaCe files was less than that in the study mentioned above.

In the present study, the results did not show any differences between the BioRaCe and Mtwo files at 3-mm level. These findings might be attributed to the lack of any severe curvature in the coronal third (3-mm level) of canal. In our study, the results at 6-mm level revealed that BioRaCe file was different from the two other groups, *i.e.* K-Flexofile and Mtwo files, with no significant differences between Mtwo and K-Flexofile. These results might be attributed to the physical features of BioRaCe instruments and the method of preparation applied in this system. In the BioRaCe group, crown-down technique was applied, but in Mtwo and K-Flexofile groups, the files were used in a single-length technique.

In the present study, there were no significant differences in canal transportation at 9-mm level between Mtwo and BioRaCe files, which might be attributed to structural properties of these files. The size and alternating cutting edges of BioRaCe files result in apical preparation up to a proper size without any increase in the number of files used. Despite differences in size and taper, the number of contact points with canal walls decreases. Therefore, the stress on file tip decreases during their feeding into the canal to the WL [17]. Indeed, the cross-sectional design of Mtwo files is S-shaped with two cutting almost perpendicular edges. This configuration results in an increase in file flexibility and its centering ability [6, 7, 32].

## Conclusion

Under the limitations of the present *in vitro* study, it can be concluded that BioRaCe and Mtwo files are suitable for canal preparation to greater apical sizes provided that the recommended sequences are observed.
